# Prevalence of suicide ideation among HIV/AIDS patients in China: A systematic review and meta-analysis

**DOI:** 10.3389/fpubh.2023.1082521

**Published:** 2023-02-23

**Authors:** Shiming Li, Shui Yu, Queping Yang, Jieyun Yin, Haohao Zhu, Ying Jiang, Yingying Ji

**Affiliations:** ^1^The Affiliated Mental Health Center of Jiangnan University, Wuxi Central Rehabilitation Hospital, Wuxi, Jiangsu, China; ^2^School of Public Health, Medical College of Soochow University, Suzhou, Jiangsu, China

**Keywords:** acquired immunodeficiency syndrome, HIV/AIDS, suicide ideation, meta-analysis, prevalence

## Abstract

**Objective:**

A systematic review and meta-analysis was performed to evaluate the prevalence of suicide ideation among HIV/AIDS patients in China.

**Methods:**

Systematic search of CNKI, Wanfang, China biology medicine database, Weipu, EMBASE, Web of science and PubMed for studies related to the suicide ideation of HIV/AIDS patients. The incidence of suicide ideation of HIV / AIDS patients in China was investigated by meta-analysis.

**Results:**

A total of 16 studies were included (*n* = 6,174). The incidence of suicidal ideation in HIV/AIDS patients was 30.6% (95%CI: 21.4–39.9%). The results of subgroup analysis showed that the incidence of suicidal ideation in male was 36.1%, which was higher than that in female (32.8%), homosexual patients (39.7%) higher than heterosexual patients (27.1%), 2013–2021 survey (35.2%) higher than 2003–2012 survey (26.5%), the unmarried patients (39.6%) were higher than the married patients (34.5%), the patients diagnosed >1 year (28.4%) were higher than the patients diagnosed <1 year (27.6%), and the depression patients (34.3%) were higher than patients without depression (20.5%) and CD4 cell counts ≤200 cells/ul group (20.6%) were higher than those in >400 cells/ul group (19.8%).

**Conclusion:**

The incidence of suicide ideation in HIV/AIDS patients in China is relatively high.

## 1. Introduction

Acquired immunodeficiency syndrome (AIDS) is an infectious disease caused by Human Immunodeficiency Virus (HIV). As of December 2020, 1.053 million AIDS patients were registered and reported in China (excluding Hong Kong, Macao and Taiwan), and more than 100,000 new HIV-infected patients were reported that year (1). With the increase of AIDS patients, the mental health of AIDS patients has attracted widespread attention of the whole society. As a traumatic and stressful event, HIV infection seriously affects the physical and mental health of patients, making them more prone to depression, anxiety, and even suicide (2–4). Studies have also identified AIDS as a potential predictor of suicidal behavior, and AIDS patients have a higher incidence of suicidal ideation than the general population (5, 6). Over time, some domestic studies have found that the incidence of suicidal ideation in AIDS patients ranges from 1.03 to 57.14% (7–11). In recent decades, the implementation of prevention and control policies and economic and social development in different regions have affected the mental health status of local AIDS patients, and the differences in the sample size of different surveys and the individual differences of the survey subjects have affected the performance of various studies. Therefore, this study adopts the Meta-analysis method to comprehensively analyze the prevalence of suicidal ideation among HIV/AIDS patients in China, with the aim to provide a scientific basis for formulating relevant policies and plans.

## 2. Methods

### 2.1. Studying retrieval

Retrieval databases include Chinese databases: CNKI, China Biology Medicine Database (CBM), Wanfang and Weipu; English databases: Web of Science, PubMed and Embase. The database was searched for “AIDS” or “acquired immunodeficiency syndrome” or “HIV”, “suicide” or “suicidal ideation”, “suicide” or “suicidal ideation”, “incidence” or “prevalence” and “China” by subject headings, abstracts, titles (or article titles) and keywords for Chinese databases and MeSH vocabulary and free words for English databases, and the retrieval time was set from 2003 to 2021. Besides, the study traceability method was applied to further search.

### 2.2. Screening and data extraction

Inclusion criteria: cross-sectional study from China; age ≥ 18 years old; with clear number of samples (≥25); the basis of suicidal ideation is clearly recorded.

Exclusion criteria: repeated publication or incomplete information; unreasonable research design and statistical methods; review or expert commentary.

Studying retrieval was carried out according to the retrieval strategy, and two researchers conducted preliminary screening of the retrieved studies, and then read the full text for secondary screening to determine the included studies according to the inclusion and exclusion criteria. Studies with different opinions were judged by a third researcher. Data were extracted according to a unified table, including the first author, publication year, study area, sample source, age range, sample size, incidence of suicidal ideation, etc. Suicidal ideation was defined as the presence of suicidal thoughts or suicidal intent.

### 2.3. Quality evaluation

The quality was scored using the AHRQ (Agency for Healthcare Research and Quality) cross-sectional study quality evaluation list (including a total of 11 items) (9). 0–3 points were identified as low quality, 4–7 points were medium quality, and 8–11 points were high quality (10). Two researchers evaluated the included studies according to the AHRQ cross-sectional study quality evaluation list. If there was any inconsistency, a third person was asked to evaluate again to avoid the different quality evaluation results.

### 2.4. Statistical analysis

Stata12.0 software was used for heterogeneity test. If the heterogeneity test I^2^ < 50%, it was considered that there was moderate or low degree of heterogeneity, so the fixed effect model was used for analysis; if I^2^ ≥ 50%, the random effect model was applied for analysis. Subgroup analysis was used to evaluate differences in gender, sexual orientation, year of investigation, marital status, time of diagnosis, history of depression and CD4 cell count. Sensitivity analysis was conducted by excluding individual studies to observe changes in the results. If the results do not change much, it indicated that the results were relatively stable. Publication bias analysis was assessed using Egger's test and funnel plots. For all statistical tests, *P* < 0.05 was considered statistically significant.

## 3. Results

### 3.1. Basic features

**Figure 1:**
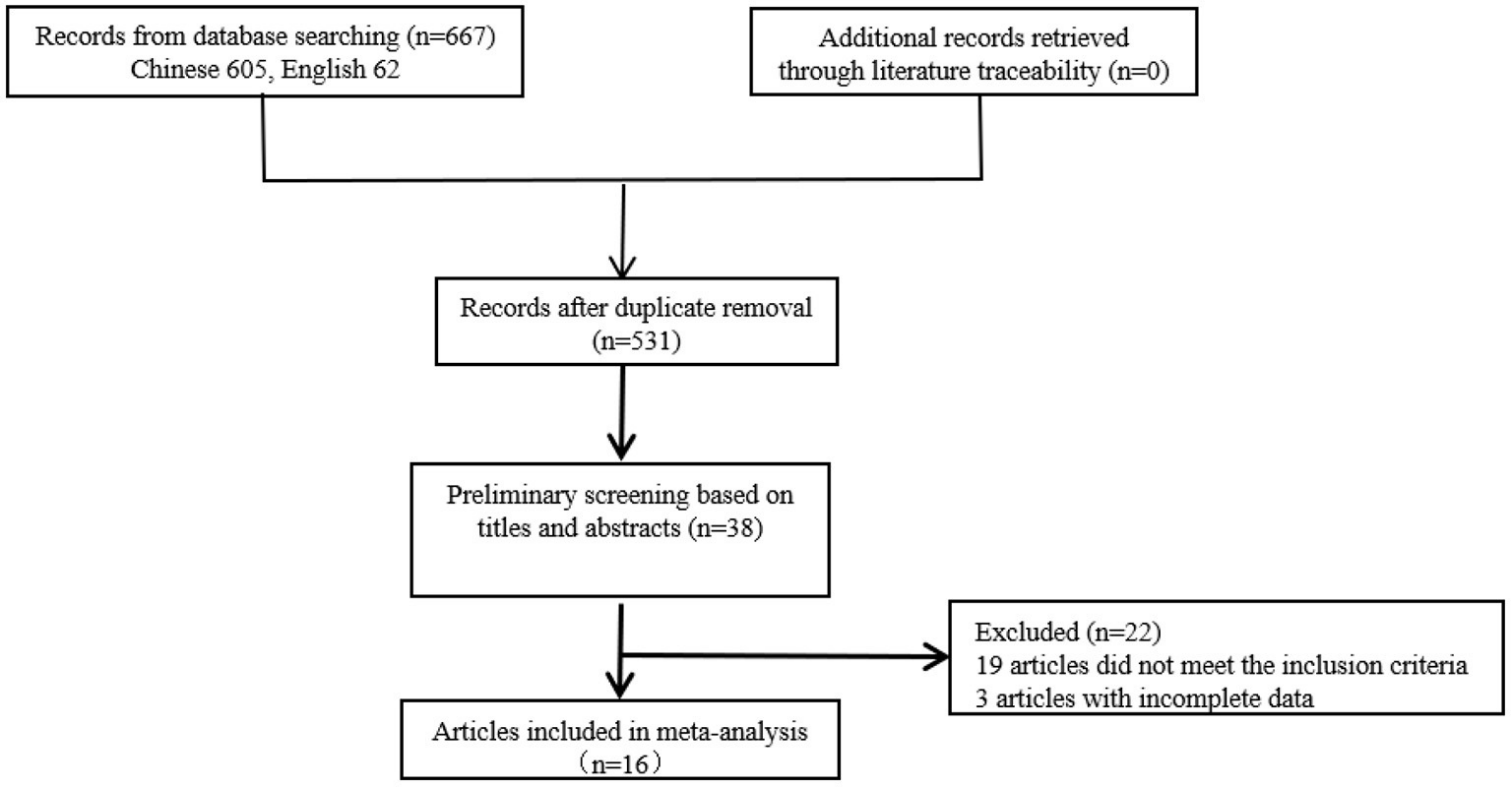
Flow chart of study selection in the meta-analysis.

The initial search yielded 667 studies (including 62 English studies), which were screened according to the inclusion and exclusion criteria. A total of 16 studies were finally selected, including 12 in Chinese and 4 in English, as shown in Figure 1. The total sample size included in this study was 6 174 people, with a variation range of 28-1 276 (*M* = 278). Among the studies included in this study, 5 are of high quality, 10 are of medium quality and 1 is of low quality (see Table 1).

**Table 1:** Characteristics of the included studies.

**No**.	**Author**	**Year**	**Region**	**Age**	**Incidence of suicidal ideation (%)**	**Quality evaluation**
1	Yu X et al. (11)	2006	Beijing, Anhui	18–50	16 (57.14)	High
2	Wu HY et al. (12)	2007	Anhui	24–67	82 (46.86)	Modern
3	Zhang XQ et al. (13)	2010	Hunan	20–52	32 (18.82)	Low
4	Lau JTF et al. (14)	2010	Central	18	60 (34.09)	Modern
5	Liang SY et al. (15)	2012	Henan	19	36 (3.19)	Modern
6	Wu DL et al. (7)	2014	Beijing	14–79	3 (1.03)	Modern
7	Qin XJ et al. (16)	2014	Guangdong	20	42 (29.17)	Modern
8	Zhang HX et al. (17)	2016	Guangzhou	21	134 (32.84)	Modern
9	Wu YL et al. (18)	2016	Anhui	18–62	57 (30.98)	Modern
10	Wang HY et al. (19)	2017	Hunan	9–72	137 (27.18)	Modern
11	Wang W et al. (20)	2018	Jiangsu	18–77	147 (31.61)	High
12	Zen CB et al. (21)	2018	Guangzhou	20–76	133 (32.36)	High
13	Liu Y et al. (22)	2019	Jiangxi	20–59	34 (40.00)	Modern
14	Liu Y et al. (23)	2019	Guangzhou	18–80	81 (17.46)	Modern
15	Yu Y et al. (8)	2021	Guangxi	18	692 (54.23)	High
16	Yang ZJ et al. (24)	2021	Shenzhen	18–65	105 (39.92)	High

### 3.2. The incidence of suicidal ideation in HIV/AIDS patients

**Figure 2:**
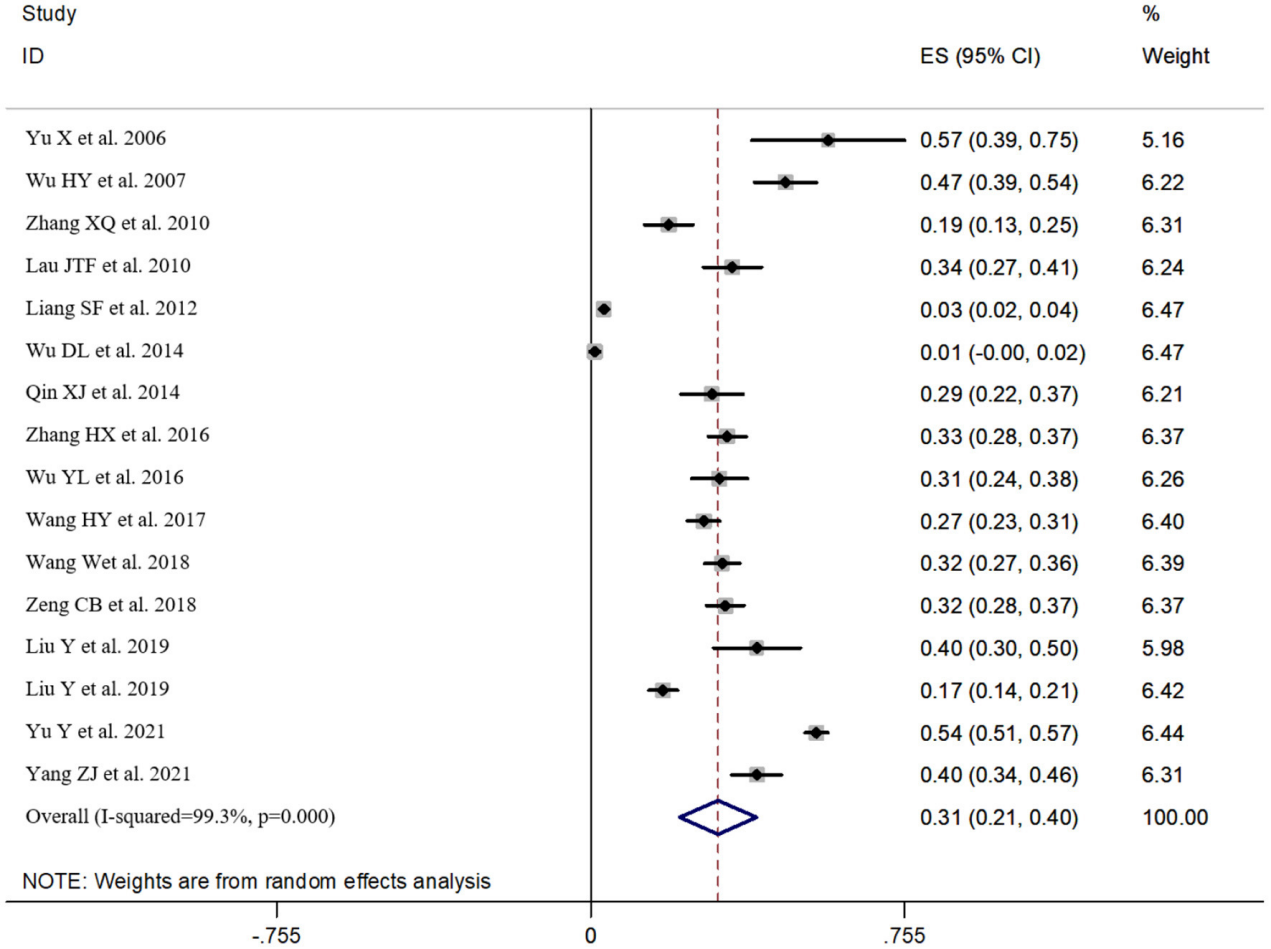
Forest map of meta-analysis of the incidence of suicidal ideation in Chinese HIV/AIDS patients.

According to the results of the heterogeneity test, there was obvious heterogeneity (I^2^ = 99.3%, *P* < 0.05). Therefore, the random effects model was used in this study. The incidence of suicidal ideation in Chinese HIV/AIDS patients was 30.6% (95%CI: 21.4~39.9%) (see Figure 2).

### 3.3. Subgroup analysis

The incidence of suicidal ideation in male was 36.1% (95%CI: 29.1–43.2%), which was higher than that in females (32.8% (95%CI: 22.0–43.6%); the incidence of suicidal ideation in homosexual patients was 39.7% (95%) CI: 32.3–47.1%), higher than that in heterosexual patients 27.1% (95% CI: 22.1–32.1%); 35.2% (95%CI: 26.8–43.5%) in 2013–2021 and 26.5% (95%CI: 18.0–35.0%) in 2003–2012, indicating the incidence of suicidal ideation with an upward trend. The incidence of suicidal ideation in unmarried patients (39.6%, 95%CI: 27.8–51.3%) was higher than that in married patients (34.5%, 95%CI: 25.1% ~43.9%); the incidence in patients diagnosed >1 year was 28.4% (95%CI: 23.9% ~33.0%), slightly higher than that in patients <1 year (27.6% (95%CI: 95%CI: 9.3% ~45.9%). Compared with non-depressive patients (20.5%, 95%CI: 17.7% ~23.3%), depressive patients (34.3%, 95%CI: 25.1% ~43.6%) was more likely to have suicidal ideation. The incidence of suicidal ideation in the CD4 cell count ≤ 200 cells/ul group was 27.3% (95%CI:6.3–48.4%), which was slightly higher than that in the CD4 cell count >400 cells/ul group (18.2%, 95%CI:4.4–31.9%) (see Table 2).

**Table 2:** Meta-analysis of the incidence of suicidal ideation in various subgroups of Chinese HIV/AIDS patients.

**Factors**	**No**.	**Sample size**	**No. of suicidal ideation**	***I^2^* (%)**	***P***	**Pooled incidence (%)**	**95% CI**
**Gender**							
Male	10	3 509	1 442	94.4	<0.05	36.1	29.1~43.2
Female	8	577	191	87.7	<0.05	32.8	22.0~43.6
**Sexual orientation**							
Homosexual	6	859	333	80.6	<0.05	39.7	32.3~47.1
Heterosexual	6	1 086	285	68.8	<0.05	27.1	22.1~32.1
**Survey year**							
2003–2012	7	2 114	317	98.5	<0.05	26.5	18.0~35.0
2013–2021	9	4 060	1 549	96.8	<0.05	35.2	26.8~43.5
**Marital status**							
Married	7	1 281	452	92.6	<0.05	34.5	25.1~43.9
Unmarried	7	2 105	937	96.3	<0.05	39.6	27.8~51.3
**Diagnosis time**							
≤1 year	6	1 022	249	98.4	<0.05	27.6	9.3~45.9
>1 year	4	750	221	44.0	0.15	28.4	23.9~33.0
**Depression**							
Yes	5	726	346	93.9	<0.05	34.3	25.1~43.6
No	5	806	166	<0.05	0.84	20.5	17.7~23.3
**CD**_4_ **cell count**							
≤200 cells/ul	4	463	130	97.4	<0.05	27.3	6.3~48.4
>400 cells/ul	4	352	63	95.3	<0.05	18.2	4.4~31.9

### 3.4. Bias and sensitivity analysis

It was found that the symmetry was general through funnel plot, and Egger's test showed *t* = 1.08, *P* = 0.19, indicating that there was no publication bias. After excluding any studies in the sensitivity analysis, the overall results did not change significantly, confirmed the stability of the serious results.

## 4. Discussion

Suicidality and HIV/AIDS are currently two major public health issues of widespread concern, which are more prominent in developing countries (25). There are currently more than 1 million AIDS patients in China, and there will be 135,000 new HIV-infected patients in 2020. Due to the lack of awareness of the disease and discrimination in the social environment after HIV infection, most patients will have a strong sense of stigma (26), which lead to anxiety and depression (27), even extreme behaviors such as suicide (28). This study found that the incidence of suicidal ideation in HIV/AIDS patients in China was 30.6% (95%CI: 21.4–39.9%), which was significantly higher than that in the general Chinese population of 3.9% (95%CI: 2.5–6.0%) (29), but lower than Nigeria (33.6%) (5) and Ethiopia (33.6%) (30). The differences among different studies may be related to ethnicity, region, economic and cultural issues, social support and acceptance, and awareness of HIV/AIDS.

The subgroup analysis found that the incidence of suicidal ideation in males was higher than in females, and the results were consistent with the studies by Kelly B et al. (31) in Switzerland and Zen CB et al. (21) in China. However, studies by Oladeji BD et al. (32) in Nigeria and Carrieri MP (33) in the United States found that the suicide risk of female patients was higher than that of males. Some studies have also reported that suicide risk was not related to gender (34). This may be because of the difference between social backgrounds of different countries, the moral requirements for men and women are also inconsistent, the social discrimination and psychological pressure are not the same. In China, most men bear the burden of economic and family care (35). The HIV infection led to the discrimination against by society, fixed economic income will be disrupted to increase the risk of suicide (34). This study found that the incidence of suicidal ideation in HIV/AIDS patients with different sexual orientations was significantly different, with the incidence of suicidal ideation in homosexual patients was 39.7% (95%, CI: 32.3–47.1%), higher than that in heterosexual patients 27.1% (95% CI: 22.1–32.1%). Compared with heterosexuals, homosexuals suffer from external discrimination and bear heavier psychological pressure. In the general population, homosexuals have a higher suicide risk than heterosexuals, which was consistent with previous domestic studies (1). Zhang et al. (1) showed that over time, with the development of time, the number of deaths of newly discovered HIV infected patients had decreased, and the suicide rate had also decreased year by year, which is in line with the positive results since the implementation of the “four exemptions and one assistance” policy of China, which included the provision of antiviral drugs for free, free consultation and preliminary screening, free mother-infant blocking drugs and infant detection reagents for infected pregnant women, exempted school fees for orphans of AIDS patients and strengthen the publicity of AIDS prevention knowledge (36). However, this study found that the incidence of suicide ideation among people with HIV/AIDS in China in 2013–2021 was higher than that in 2003–2012, which may be because of the increased social pressure from study, life and employment, which will aggravate the appearance of self-abuse thoughts, especially for HIV/AIDS patients (20, 37). Unmarried HIV/AIDS patients were found with higher suicidal ideation than married patients. Compared with married patients, unmarried patients often lacked family support and care, which may lead to more stress, depression, anxiety and suicide ideation (16, 18). In terms of time to diagnosis, we found that the incidence of suicidal ideation in patients diagnosed >1 year was slightly higher than that in patients ≤1 year, while other studies have shown that suicidal ideation and suicidal behavior were more likely to occur soon after HIV infection was diagnosed, the incidence of suicidal ideation will be decreased with more adaptation time and social support (1, 18). The incidence of suicidal ideation in patients with a history of depression was much higher, since mental health problems can increase the risk of suicide. A domestic study confirmed that the suicide risk of AIDS patients with depression was 3.7 times that of patients without depression (38). Related reports (39) showed that depression was an independent risk factor for suicide in AIDS patients. Patients with low CD4 cell counts have a higher incidence of suicidal ideation than these with high CD4 counts. Studies have confirmed that the more the number of CD4 cells in patients, the less damage to the immune system, and the lower the incidence of suicide (40). This may be because the patients were in different disease courses, and the risk of suicide was different. Due to various infections and side effects of anti-HIV treatment, the immune system will be severely damaged, the CD4 cell count will be decreased, and the quality of life will be decreased, resulting in suicidal ideation, or even suicidal behavior (40).

The advantage of this study is that it systematically assessed the occurrence of suicidal ideation among HIV/AIDS patients in China, and provided new thinking and scientific basis for the formulation of psychological intervention policies and prevention and control priorities for AIDS patients. There were still several deficiencies in this study: (1) Due to the lack of a unified measurement scale for suicide in AIDS patients, a unified standard cannot be used to assess suicidal ideation, and there is information bias and selection bias; (2) This study also did not explore the effects of other co-morbid mental disorders and the impact of major emergencies in life on patients.

## 5. Conclusion

The incidence of suicide ideation in HIV/AIDS patients in China is relatively high. In order to effectively reduce the suicide risk of HIV/AIDS patients, relevant government departments should strengthen the level of AIDS-related knowledge and further enhance the level of social support for people with HIV/AIDS. Policy makers should strengthen the cooperation between the Centers for Disease Control and Prevention and specialized mental health medical institutions, and highlight mental health education and psychological crisis intervention services on the basis of “four exemptions and one assistance”, so as to improve the quality of life of HIVAIDS patients and reduce the risk of suicide.

## Data availability statement

The dataset generated and analyzed during the current study could be available from the corresponding authors on reasonable request.

## Author contributions

Study design: SL and HZ. Analysis and interpretation of data: YJia, QY, JY, and YJi. Drafting of the manuscript: SL and YJia. Critical revision of the manuscript: SY and HZ. Approval of the final version for publication: All authors. All authors contributed to the article and approved the submitted version.
